# A Deep Neural Network Based Model for a Kind of Magnetorheological Dampers

**DOI:** 10.3390/s19061333

**Published:** 2019-03-17

**Authors:** Carlos A. Duchanoy, Marco A. Moreno-Armendáriz, Juan C. Moreno-Torres, Carlos A. Cruz-Villar

**Affiliations:** 1 Cátedra CONACyT, Instituto Politécnico Nacional, Centro de Investigación en Computación, Av. Juan de Dios Bátiz s/n, Ciudad de México 07738, Mexico; 2 Instituto Politécnico Nacional, Centro de Investigación en Computación, Av. Juan de Dios Bátiz s/n, Ciudad de México 07738, Mexico; mam_armendariz@cic.ipn.mx (M.A.M.-A.); juancmt27@hotmail.com (J.C.M.-T.); 3 Electrical Engineering Department, Mechatronics Section, CINVESTAV-IPN, Mexico 07360, Mexico; cacruz@cinvestav.mx

**Keywords:** automotive applications, computational modeling, neural networks

## Abstract

In this paper, a deep neural network based model for a set of small-scale magnetorheological dampers (MRD) is developed where relevant parameters that have a physical meaning are inputs to the model. An experimental platform and a 3D-printing rapid prototyping facility provided a set of different conditions including MRD filled with two different MR fluids, which were used to train a Deep Neural Network (DNN), which is the core of the proposed model. Testing results indicate the model could forecast the hysteretic response of magnetorheological dampers for different load conditions and various physical configurations.

## 1. Introduction

Magnetorheological dampers have been an area of active research over the last 20 years. Applications include civil engineering [1], mitigation of seismic effects [2], automotive suspensions [3], seat comfort improvement [4], washing machines [5] and advanced prosthetics [6]. These applications have met with success, as model-based control techniques have been developed on the ground of models that properly capture the hysteretic behavior of MRD.

MRD models can be classified into parametric and non-parametric models. Bingham models, biviscous models, and Bouc–Wen models are the most common parametric models used [7]. Most of the non-parametric models are based on polynomial approximation [8], neural networks [9], fuzzy logic [10], fuzzy neural network and particle swarm optimization [11].

In parametric models, parameters have a physical meaning such as damping, friction or stiffness. However, none of the above models include magnetorheological damper parameters such as piston geometry, gap area, etc. This kind of parameters is more design-oriented instead of control-oriented of an MR damper. Model-based design of MR dampers has been based on quasi-static assumptions [12], finite element models [13], as well as on combinations of theoretical model and finite element analysis [14,15]. Quasi-static models do not completely describe the nonlinear behavior of the force-velocity relationship. On the other hand, although finite element models (FEM) provide high accuracy, it comes at the cost of high computing time, this fact can render the design process inefficient.

Other interesting applications where neural networks (NN) have been applied in the modeling of complex problems are as follows. In [16], the authors proposed a new probabilistic power-flow model based on radial-basis neural network (RBFNN). This novel model improves the computer time to solve this complex task and its efficiency was proved by testing on different microgrids including wind generation and photovoltaic units, plug-in hybrid electric vehicles and uncertain loads. In [17], an Adaptive Neuro Fuzzy Inference System (ANFIS) is used to design a super sliding mode controller for body vibration control in active quarter car system.Finally, a fault detection via neural networks for photovoltaic module under partially shaded conditions is introduced in [18]. The NN can estimate the output photovoltaic current and voltage under variable working conditions. The results show that the NN accurately detects the shading effect on the photovoltaic module. Other significant contributions in this area can be found in [19,20,21,22,23,24,25,26].

In this paper, a novel modeling methodology that combines first-principles modeling and non-parametric modeling techniques to represent the MR damper is presented. First-principle models are used to model the spring and the magnetic circuit. From properties of the magnetorheological fluid, a parametric fluid model is obtained. Hydraulic force of the damper is modeled using a hybrid model, the viscous component of the force is modeled via a first-principles model, and the magnetic component of the hydraulic force and hysteresis are modeled by a nonparametric model through deep learning [27,28]. The main rationale for the use of deep neural networks [29] stems from the recognition that shallow neural networks are well suited to model a specific MR damper. However, in this work, it is not from a particular MR damper whose model is to be obtained, but from a family of MR dampers.

From the perspective of machine learning, one of the main challenges in implementing a single model for a family of MRDs that includes geometry parameters lies in the generation of enough experimental data to train a deep neural network. To this end, a 3D printing facility and an experimental platform were used to generate such dataset, where different geometry parameters and two different MR fluids were considered.

The proposed model was created to perform an optimal design, which, unlike the control models, should be able to reflect the change in the behavior of the shock absorber with respect to the physical design variables, such as the type of oil or the dimensions of the hydraulic system, among others and also to changes in the magnetic field and speed. In contrast, traditional models can only react to changes in the last two variables mentioned. With this objective in mind, it was necessary to grow the complexity of the model, obtaining in return a model with more capabilities than what is reported in the state of the art.

The rest of the paper is organized as follows. Section 2 states the relationship between the independent and dependent variables for the proposed model. Section 3 describes the proposal. Then, the experimental setup to acquire the training and validation sets for the MR damper modeling, a comparison of the performance of a shallow and a deep neural network for modeling MRDs and a discussion of the resulting models including the validation and error analysis is presented in Section 4. Section 5 concludes the paper.

## 2. Case Study: Magnetorheological Damper

**Figure 1:**
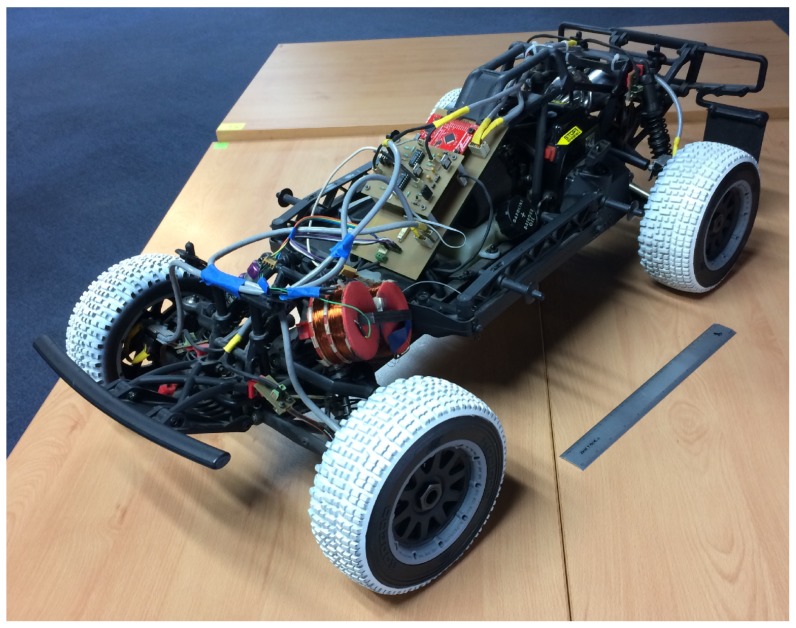
Scale vehicle for the case study.

**Figure 2:**
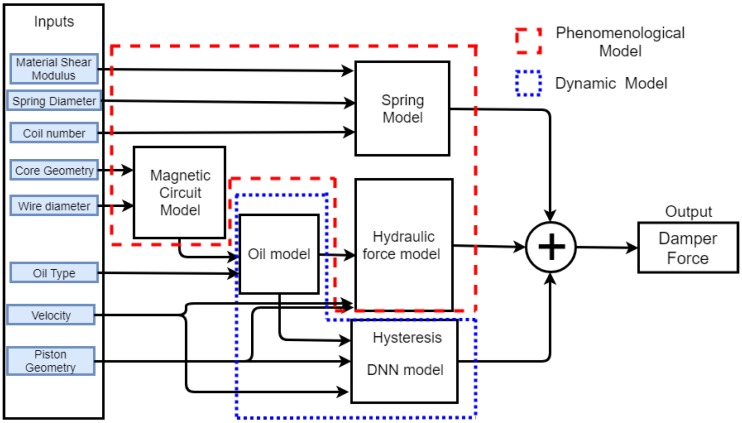
Block diagram for MR damper modeling.

As a case study, we selected a small size magnetorheological damper for a scale vehicle shown in Figure 1, which was tested at low velocities in the pre-yield region operating in valve mode. The damper force has three components: the spring force, the hydraulic viscous force and the biviscous hysteretic force. In this modeling approach, the first-principle dynamic models for the magnetic circuit, the mechanical spring and the viscous component of the MR force is used. The oil behavior is modeled via a parametric model obtained from characteristic curves provided by the fluid manufacturer. The main interest of this work lies in obtaining a model able to capture the effect of different MR fluids and different piston geometries; the fluid hysteretic behavior is modeled by a non-parametric model through a Deep Neural Network (DNN), as shown in Figure 2.

### 2.1. Spring Model

The helical spring is modeled by the Hooke’s law and, according to Saverasi et al. [30], its stiffness $K_{t}$ is given by Equation (1), where *R* is the helix radius, *d* is the wire diameter, *G* is the rigidity modulus and *n* is the number of active coils.

(1) $$K_{t}=\frac{Gd^{4}}{32R^{3}n}$$

### 2.2. Magnetic Circuit Model

**Figure 3:**
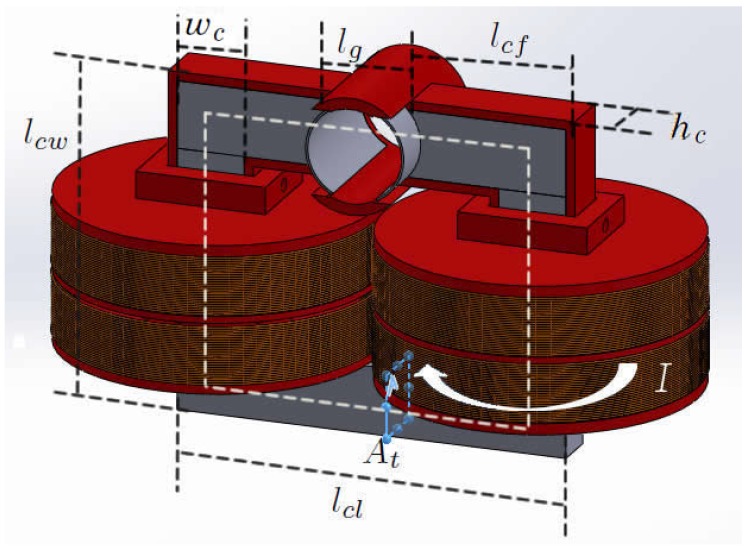
Magnetic circuit.

The proposed magnetic circuit, shown in Figure 3, can be represented by a simpler circuit. The full length of the core $l_{c}$ is given by Equation (2) and the transversal area $A_{t}$ is given by Equation (3), where $N_{t}$ is the number of turns and $l_{g}$ is the air gap length.

(2) $$l_{c}=l_{cw}+l_{cl}+l_{cf}$$

(3) $$A_{t}=w_{c}\cdot h_{c}$$

In a magnetic circuit, the magnetic field must overcome the material’s reluctance, which must be defined for each segment of the different materials in the magnetic circuit. The core material $R_{c}$ and air gap $R_{g}$ reluctances are given by Equations (4) and (5), respectively, where $\mu_{c}$ is the core permeability coefficient and $\mu_{0}$ is the air permeability coefficient.

(4) $$\begin{array}{ccc} R_{c} & = & \frac{l_{c}}{\mu_{c}A_{t}} \end{array}$$

(5) $$\begin{array}{ccc} R_{g} & = & \frac{l_{g}}{\mu_{0}A_{t}} \end{array}$$

It is assumed that the magnetic flux ϕ is continuous across the different cross sections. Moreover, magnetic losses such as fringe flux, leakage flux, and core loss can be approximated by a single resistance. Therefore, the magnetic flux is given by Equation (6).

(6) $$\phi =\frac{N_{t}I}{R_{c}+R_{g}}$$

The magnetic field $B_{c}$ can be obtained using Equation (7).

(7) $$B_{c}=\frac{\phi }{A_{t}}$$

Special attention must be paid to the core magnetic flux density due to magnetic material saturation, which may lead to a change of its properties. The air gap, instead, is not saturable, because of the linear magnetization curve of this material. In saturation, the magnetic field output $B_{o}$ is given by Equation (8), where $B_{sat}$ is the core magnetic field at saturation.

(8) $$B_{o}=\frac{B_{sat}B_{c}}{B_{c}+B_{sat}}$$

### 2.3. Oil Model

A magnetorheological oil has metallic magnetically polarized particles suspended in a carrier fluid such as silicon or mineral. The main characteristic of a magnetorheological oil is that its physical properties vary according to the magnetic field $B_{out}$ applied to the fluid. When the fluid is exposed to a magnetic field the metallic particles align themselves along the magnetic flux lines in a matter of milliseconds. On the other hand, in the absence of a magnetic field, the fluid behaves as an ordinary oil.

To know the behavior of the magnetorheological fluid, the supplier provides a series of graphics that describe the behavior of the magnetorheological fluid when exposed to a magnetic field [31]. The graphics for each magnetorheological fluid were approximated by a polynomial given in Equation (9). Constants $c_{0}$–$c_{4}$ for each material are presented in Table 1.

(9) $$P\left(x\right)=c_{0}+c_{1}x+c_{2}x^{2}+c_{3}x^{3}+c_{4}x^{4}$$

From the properties of the magnetorheological fluid, two polynomials for each type of oil can be determined. The first polynomial, in Equation (10), gives the magnetic field intensity *H* as a function of the magnetic flux density $B_{out}$ in the ferromagnetic material. The second polynomial, in Equation (11), gives the yield stress $T_{y}$ as a function of the magnetic field intensity *H*.

(10) $$\begin{array}{ccc} H(B_{o}) & = & c_{0}+c_{1}B_{o}+c_{2}B_{o}^{2}+c_{3}B_{o}^{3}+c_{4}B_{o}^{4} \end{array}$$

(11) $$\begin{array}{ccc} T_{y}\left(H\right) & = & c_{0}+c_{1}H+c_{2}H^{2}+c_{3}H^{3} \end{array}$$

### 2.4. Damper Hydraulic Force Model

The damper is used in pressure-driven flow mode; this mode can provide large magnitude damping forces and large damper displacements. For these reasons, this type of damper is commonly used for automotive suspensions.

In [32], a model representing the force-velocity behavior of the magnetoreological damper in the pressure-driven flow mode with an annular conduit is presented. Such a model is based on the Navier–Stokes equations. Consequently, the force developed by a shock absorber operating in a pressure-driven mode is given by Equation (12), where $F_{\eta }$ is the viscous component of the force and $F_{T_{y}}$ is the magnetic component of the force.

(12) $$F=F_{\eta }+F_{T_{y}}$$

**Figure 4:**
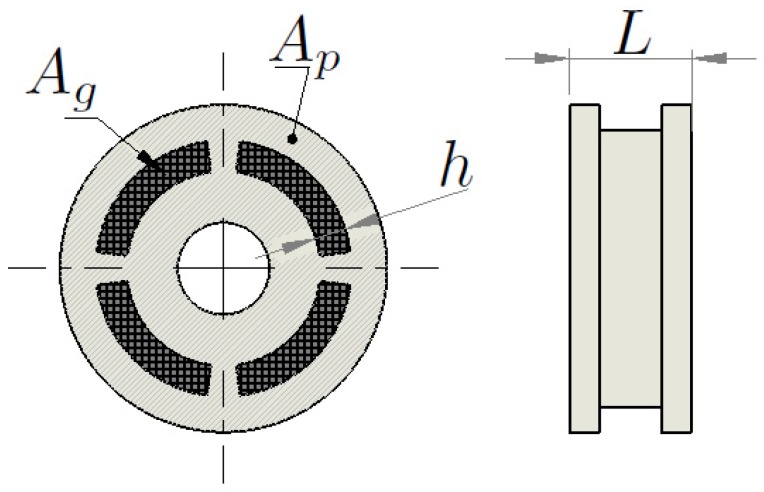
Piston head geometry.

The viscous component of the force $F_{\eta }$ is given by Equation (13), where η is the fluid viscosity, $\dot{x}$ is the piston head velocity, $A_{g}$ is the valve gap area, *h* is the gap width and $A_{rm}$ is the mean area of the gap, given by Equation (14), where $A_{p}$ is the head piston area (see Figure 4). The magnetic component of the force $F_{T_{y}}$ is given by Equation (15), where *L* is the gap length, $T_{y}$ is the yield stress and *c* is a function of the flow velocity profile as defined by Guglielmino et al. [32].

(13) $$\begin{array}{ccc} F_{\eta } & = & \frac{12\eta A_{rm}\dot{x}}{A_{g}h^{2}} \end{array}$$

(14) $$\begin{array}{ccc} A_{rm} & = & A_{p}+\frac{A_{g}}{2} \end{array}$$

(15) $$\begin{array}{ccc} F_{T_{y}} & = & \frac{cT_{y}A_{p}}{h}L \end{array}$$

As mentioned above, *c* has been defined as a function of the flow velocity profile, which is a linear approximation of the typical behavior of a magnetic hysteresis in the yield region. Since is very important to obtain an accurate model of the MR damper, the magnetorheological force component of the damper $F_{T_{y}}$ is replaced by a non-parametric model capable of representing the damper hysteresis in terms of geometry parameters. Therefore, the complete model will be suitable for both design and control of the MR damper.

## 3. Hysteresis Deep Neural Network Model

Phenomenological models rely on knowing the function that describes the nonlinear characteristics of the damper hysteresis. This kind of modeling is commonly made through experimental measures of a given physical damper. Such a methodology is useful for obtaining a phenomenological model for the hysteresis behavior of a particular damper and it is typically used for control purposes. Nevertheless, in this work, a phenomenological model useful for both design and control purposes is sought. Therefore, the model must be able to represent the hysteretic behavior of the damper for a range of different physical parameters. This cannot be achieved by measuring the behavior of one single damper, thus a range of different dampers to generate a model for a family of MR dampers was measured.

To develop a phenomenological model for the family of shock absorbers, it is necessary to measure the behavior of multiple representative devices, trying to cover, as far as possible, scattered examples through all possible devices in the family. This was possible thanks to the increase in quality and low cost of additive manufacturing, commonly known as 3D printing, which has made plausible the quick and economical manufacture of a high rate of damper prototypes.

Representing the behavior of a family of shock absorbers presents a challenge for non-parametric models, mainly because a small change in physical characteristics generates a large change in the dynamic behavior of the system, thus considerably increasing the complexity of the model. Conventional nonparametric models such as classical neural networks are capable of learning data representation directly related to the output, but are limited in their ability to extract relations from data in their raw form. In contrast, deep learning is capable of learning representations of data with multiple levels of abstraction from raw data [27]. This fact is the main motivation to use DNN, which have proven capable of dealing with nonlinear multivariate regression problems [33].

## 4. Modeling Results

In this section, a detailed description of the hysteresis DNN model is presented. In addition, the MR damper family, the dataset generated to train the model, the neural network design process and the training procedure are described.

### 4.1. Magnetorheological Damper Family

Model complexity grows proportionally to the size of the damper family. Such particular family of dampers can be classified as small-scale monotube MR dampers operating in valve mode. To avoid major changes in the scale vehicle, the general dimension of the suspension system was kept, thus preserving the tube dimensions and limiting the changes to the piston head geometry and the oil type. Two variables of the piston head geometry, the valve gap and the piston length, were considered. The valve gap was limited by the manufacturing process within a maximum of 2.5 mm to a minimum of 0.5 mm. The piston length was limited by the suspension travel allowing a maximum of 12 mm and a minimum of 3 mm. The MR fluid was limited to off-the-shell availability; therefore, we selected two fluids: MRF-140CG^®^ and MRF-122EG^®^ (Table 1). Eleven MR damper prototypes were built and measured, and each prototype was tested for ten different magnetic fields for each of the two magnetorheological fluids.

### 4.2. Dataset Analysis

**Figure 5:**
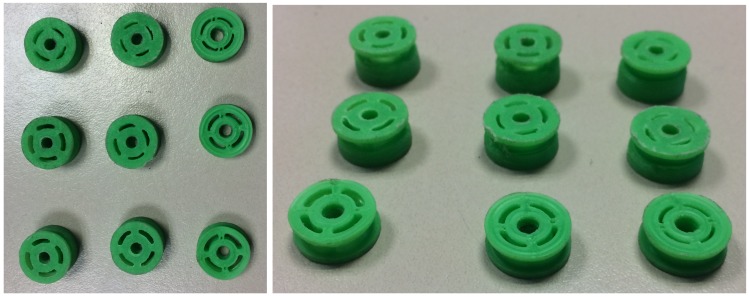
3D printed piston heads.

**Figure 6:**
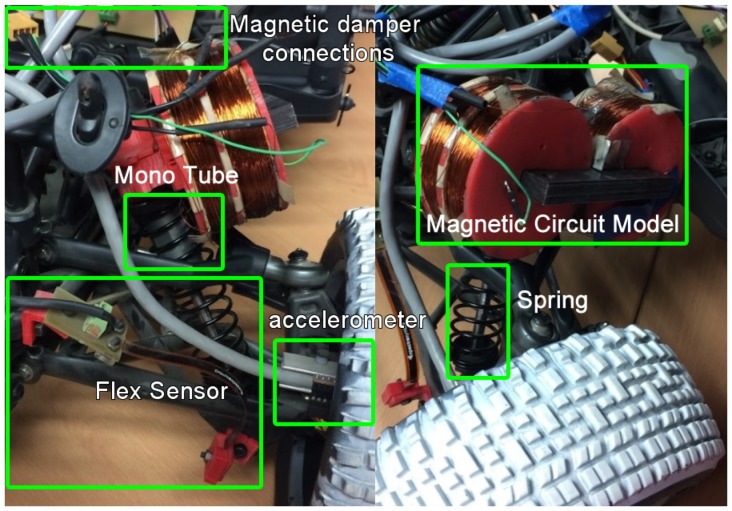
Physical components of the proposed model.

A total of 880 experiments were performed using the damper family above described. Each experiment consisted of a full cycle of 120 points on average. In Figure 5 a picture of nine 3D printed piston heads is shown. The experiments were performed using a test bench, which was instrumented to measure force, velocity and damper displacement. In Figure 6, the vehicle instrumentation is shown.

When carrying out construction and assembly of each the aforementioned prototypes, it was difficult to control all conditions to guarantee uniformity among the prototypes. These conditions should be considered since they negatively impact the training of the phenomenological model. The first of these conditions was the manufacturing tolerance; components were built with an additive manufacturing system that has a precision of 0.3 mm. Therefore, variations due to the manufacturing tolerance were significant and reflected in the behavior of the magnetorheological damper. Second, surface roughness of the finishes, which may vary by multiple conditions such as the quality of the plastic filament and the environment temperature, was of interest in our case since the piston head Was in direct contact with the wall of the cylinder and roughness influences the friction, thus having an important effect in the final force. The third was the assembly process, in particular the filling process with the magnetorheological fluid. Although it was intended to guarantee that the system had the same amount of fluid, it actually changes each time we filled the damper. Due to these conditions, the training dataset had measurements with small variations for a single input condition; thus, the construction of each prototype was carried out twice and measurements were made for two different assemblies. Therefore, four experiments were obtained per prototype. The additional measurements had the intention of allowing the system to be capable of considering the manufacturing and assembly variations as part of the prediction of the model.

### 4.3. Neural Network Design

A neural network is constituted by a set of parameters and hyperparameters. The parameters are the weights and bias of the neural network (called internal variables); these values are adjusted by an optimization process to fit data. The hyperparameters that are defined by the problem are the number of inputs and outputs. The network structure hyperparameters are the number of hidden layers and activation functions while the training algorithm hyperparameters are optimization algorithm, learning rate, batch size, cost function, and regularization techniques.

**Figure 7:**
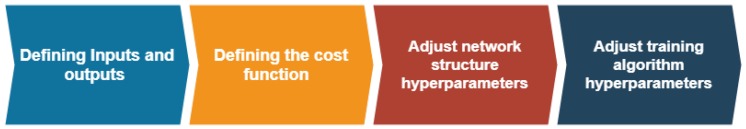
Test bench for magnetorheological damper.

The process of selecting a suitable configuration of hyperparameters is called neural network design. A process consisting of four steps shown in Figure 7 was established to design a neural network able to model the hysteresis behavior accurately.

The first step is to define the inputs and outputs of the deep neural network from the problem statement. In this case, the output was fixed as the magnetorheological force component of the damper $F_{T_{y}}$, and the inputs were chosen to be the design parameters directly related to the magnetic force performance and the physical variables that determine its behavior. Physical parameters of the damper that directly impact on the hysteresis behavior are piston length *L*, gap width *h*, gap area $A_{g}$, and piston area $A_{p}$; the parameters that describe the oil performance are viscosity η and yield stress $T_{y}$; and the physical parameters that impact the damper functionality are piston velocity $\dot{x}$ and displacement *x*.

Once the inputs and outputs are selected, it is necessary to determine a cost function suitable for the problem in the second step. The Mean Squared Error (MSE) [34] has been widely used in regression problems and the gradient of MSE loss is high for larger loss values and decreases as loss approaches to zero allowing the regressor to become more precise at the end of training. For these reasons, the MSE was selected as the cost function.

The process of selecting a suitable set of neural network hyperparameters is a special kind of optimization where the problem is nested within another, called bilevel optimization [35]. The outer problem is selecting the neural network hyperparameters that allow solving the inner problem of selecting the best parameters to approximate the model. The hierarchy of the problem generates the need to solve the inner optimization problem (training) for each hyperparameter configuration to test its performance. Therefore, to analyze the performance of the different network structures, the MSE was also used as a cost function, because it has performed well in other regression problems [9,36]. A batch size of 100 and Adam optimizer [37] were used as a pre-training configuration to solve the inner problem. Each element is described in Section 4.4.

In the third step, it is necessary to adjust the network structure starting by setting the number of hidden layers and units. Determining the number of hidden layers is a critical part in deciding the neural network architecture. These layers have a tremendous influence on the neural network overall behavior. For these reasons, the number of hidden layers and the number of units in each layer must be carefully selected.

Using a small number of layers and neurons may result in a network incapable of learning the relations between the inputs and outputs. This phenomenon is called underfitting. In contrast, using too many hidden layers and neurons will result in a network difficult to train, which may result in overfitting. Cybenko stated in the universal approximation theorem [38] that a single hidden layer network can approximate any continuous function. However, this theorem does not state how difficult it can be to train a neural network to approximate a function. In practice, all the research performed in deep learning have shown that a neural network with a higher number of hidden layers has a better performance in complex problems.

**Figure 8:**
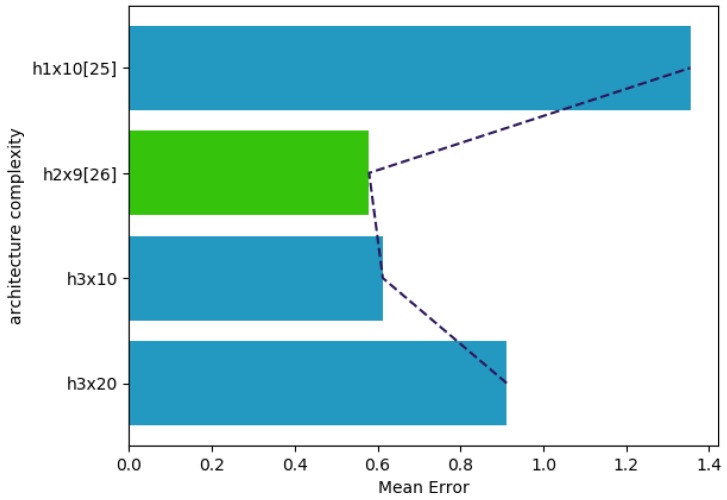
Architecture complexity for modeling a single damper configuration.

Most existing methodologies determine the number of hidden layers and neurons through a trial and error rule [39]. In this case, a grid search was selected [40] to find a suitable network structure. As the starting point, the architectures that have been used to model a single magnetorheological damper were selected [9,36] and an analysis of the literature architectures with the same conditions considering exclusively one magnetorheological configuration was performed. The results of these architectures are shown in Figure 8, where it can be seen that the best performance is obtained by a shallow architecture with two hidden layers of nine neurons each (h2x9).

**Figure 9:**
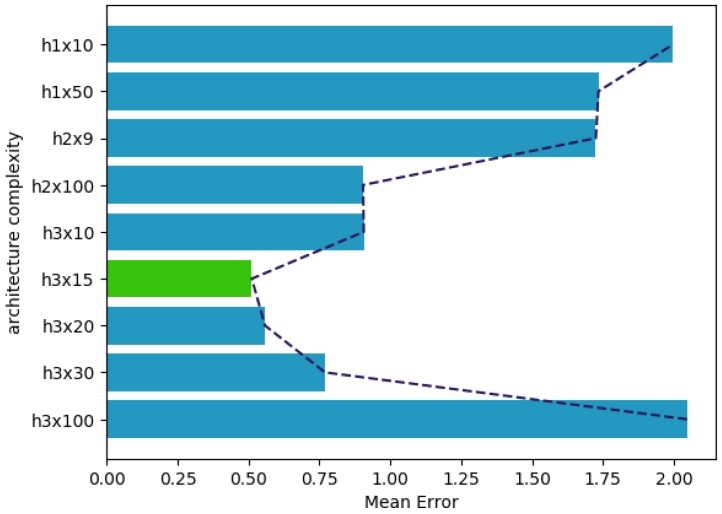
Architecture complexity for modeling a family of dampers.

Selecting these shallow networks as the starting point, the next step is to find a network architecture capable of modeling an entire family of dampers. This problem is considerably more complex than modeling a single damper, which motivated us to experiment with deeper architectures. The performance of the model generated by the different architectures is shown in Figure 9. The architecture that obtained better performance was a deep architecture with three hidden layers of 15 neurons each (h3x15).

**Figure 10:**
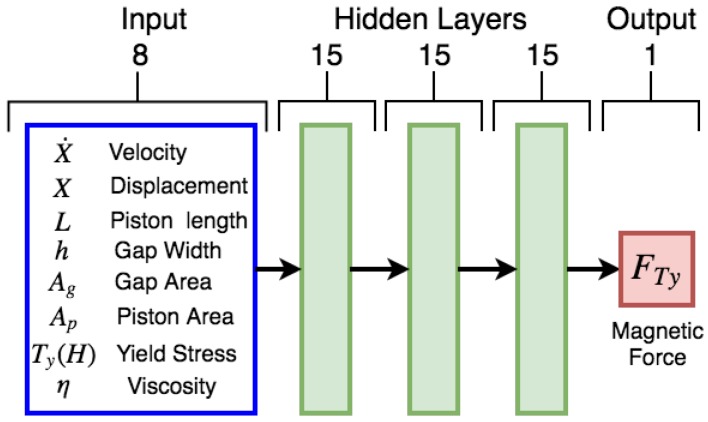
Deep neural network for hysteresis modeling.

The selected neural network architecture is a deep multilayer perceptron with eight inputs, three hidden layers of fifteen neurons each and an output layer of a single neuron, as shown in Figure 10. In [41], a similar study of the number of neurons is presented, but with a fixed number of layers for a planetary gearbox fault. Each hidden layer uses the rectified linear function (ReLU) [42] as the activation function. ReLU was selected because it reduces the likelihood of the vanishing of the gradient in deep architectures.

During the experimentation for determining the best neural network structure for modeling a family of dampers, it was found that the size of the network should be increased when the complexity of the model is raised. The model complexity is affected by the number of inputs in particular when the output has a high sensitivity to a small change in a particular input. In this particular problem, the model had a high sensitivity to the oil type input.

**Figure 11:**
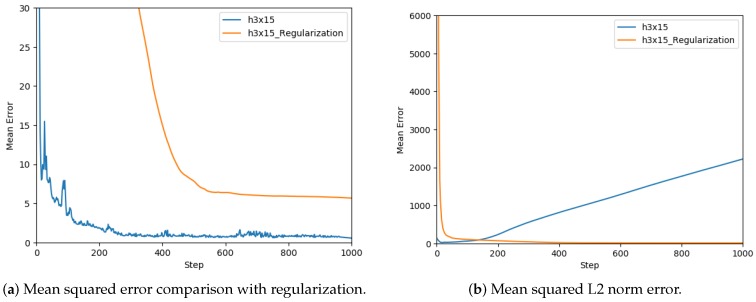
Regularization and L2 analysis.

After selecting a network architecture, the need to use regularization techniques was analyzed, thus the performance of the network using the L2 norm [43] was tested. In Figure 11a, the comparison between the performance of the network with regularization is shown. The use of L2 norm has the objective of reducing weights, shown in Figure 11b, and thus making the network more robust to losing any individual connection in the network. In this case, the regularization deteriorated the performance of the model, thus it was not appropriate to use it for training.

The final step is to adjust the training algorithm hyperparameters, which are the optimization method, the learning rate, and the batch size. Commonly, a variant of stochastic gradient descent (SGD) [44] is used as an optimization method for training complex DNN. In this case, the Adam optimizer was selected, which is capable of adjusting the learning rate at each epoch [37]. The optimization methods based on SGD update the weights form a gradient estimated from a fraction of the training data called mini batch; the size of this fraction is determined by the batch size. The learning rate is used to determine the magnitude of the influence of the mini batch gradient during the weights update. The learning rate and batch size were adjusted using a grid search, selecting a batch size of 20 and a learning rate of $1e^{-4}$ to train this model. The number of epochs was adjusted heuristically.

### 4.4. Training Hyperparameters

The data were divided into two sets: the measures of eight prototypes for training and the three remaining prototypes for testing and validation. As a result, 76,800 samples for training and 28,800 for testing and validation were used.

Adam was the selected optimization algorithm, which has the advantage of adjusting the learning rate by combining the benefits of the adaptive Gradient algorithm (AdaGrad) [45] and the root mean square propagation (RMSProp) [46]. Adam is a popular algorithm in deep learning because it has been well suited to a wide range of optimization problems [37].

**Figure 12:**
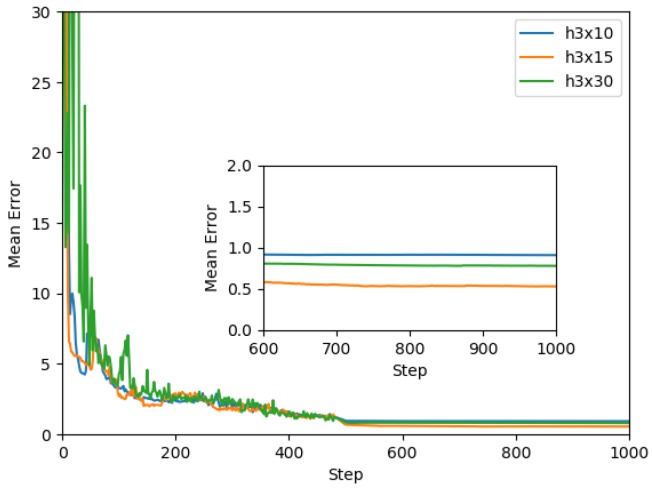
Mean squared error vs. epoch.

The model was trained for all proposed architectures discussed in the previous section. In Figure 12, a graph of the mean squared error vs. epoch of the best three models is presented. It was observed that the most complex architecture (h3x30) presented a noisier error graph and stopped learning with a larger final error than the other architectures. In comparison, the smallest architecture (h3x10) had little noise during its training but it stopped learning with the largest final error. The best model was the h3x15 architecture with a complexity between the other two models and its final error was the smallest one. Noise monitoring during the training process was very helpful in determining what changes should be made to the architecture to improve its behavior.

The neural network architecture was implemented using Tensorflow [47]. The neural network code and the dataset are available in the GitHub repository [48].

### 4.5. Validation

Most magnetorheological (MR) damper models are made for a specific objective such as response analysis, optimal control development or design of new damper configurations. The magnetorheological damper has gained attention in automotive and structural engineering applications where rapid response is critical [49]. The response analysis models are developed to evaluate the damper performance in this aspect these models do not need to detail the intrinsic nonlinear nature of MR dampers and only use a linear representation. To develop suitable control algorithms for MR dampers, it is necessary to use models that are capable of accurately reproducing the nonlinear behavior and hysteretic response of the MR damper. In [50], a comparison of the main models for control is presented. For the optimization task, the MR damper models must be capable of approximating the behavior in function of the design parameters, otherwise they are not suitable. In Table 2, a comparison of the proposed model with the models in the literature [7] is presented. The new model is unique with the ability to model the hysteresis as a nonlinear phenomenon and be used for the tasks of control, design, and optimization.

**Figure 13:**
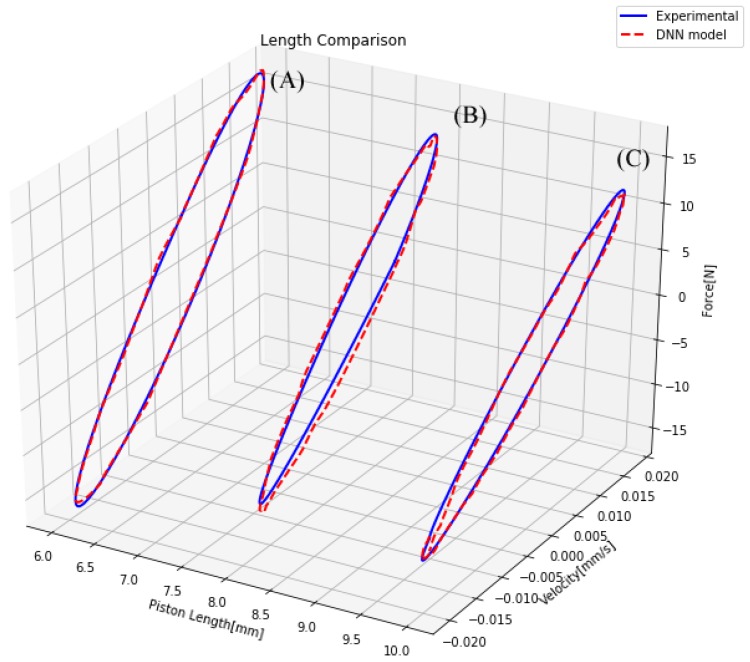
Comparison of a damper with different piston length.

**Figure 14:**
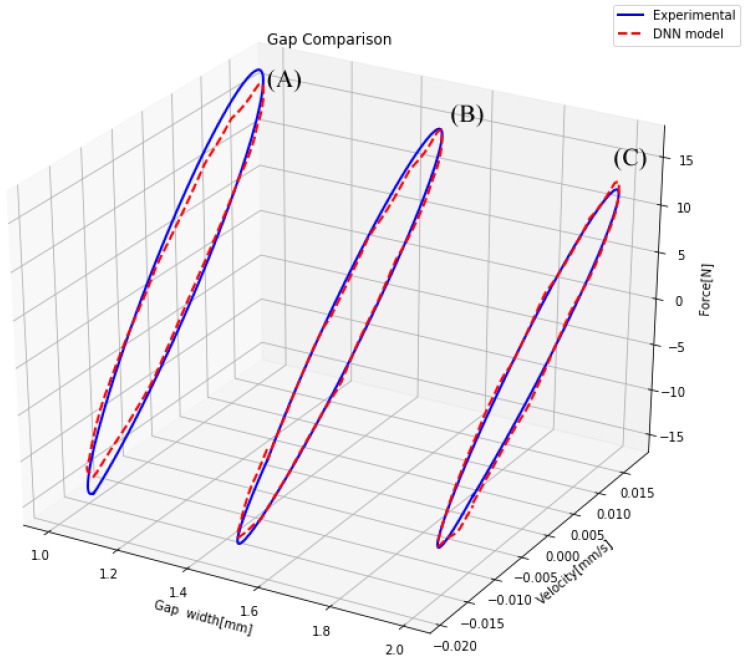
Comparison of a damper with different gap area.

To verify the accuracy of the novel model, it was necessary to test its similarity to the actual measurements. Therefore, several comparisons between the actual measurements and the model output for different sets of inputs were performed. First, the performance of the model against changes in the piston geometry was tested. This experiment was separated into two cases: a change in the piston head length and a change in the piston gap width. In both experiments, only one of these inputs was modified and the rest remained fixed. These conditions allowed isolating the performance of the model to the change of each parameter independently. In Figure 13, three different lengths are considered, and it can be observed that the model could perform well with piston length changes. In Figure 14, three different gap areas are considered. One fact that affected the performance of the model in this particular case was the manufacture precision of the 3D printer, which performed poorly when the gap width was small. This manufacturing error generated noise measures in most small gap cases, as shown in Figure 14A.

**Figure 15:**
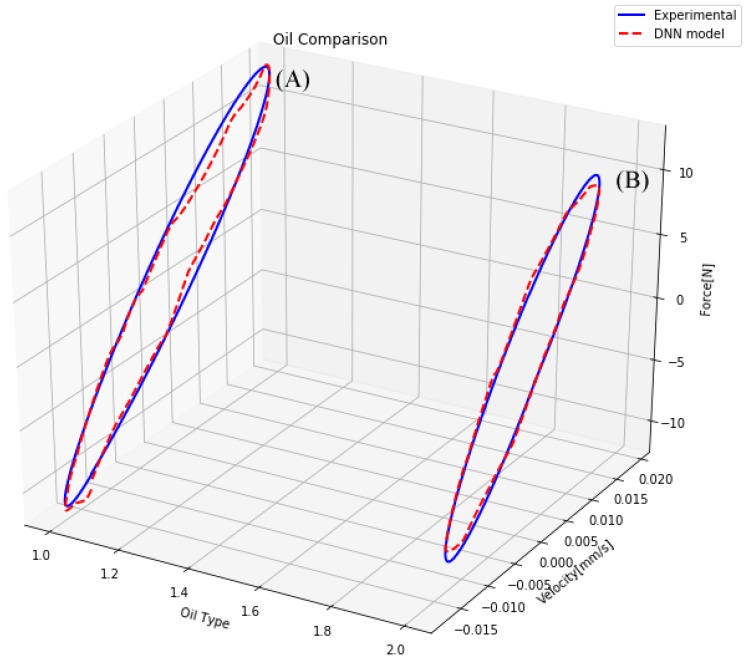
Comparison of a damper with different magnetorheological oil type.

The second factor considered was the oil type design parameter. It worked as a selector since only one indicator number was changed. However, it radically changed the behavior of the magnetorheological damper. This design parameter was particularly challenging to model because a small change in an input drastically changed the behavior of the damper. To analyze if the model could represent a change in the oil type, a comparison of the same shock absorber filled with two different oils is shown in Figure 15. Considering the difficulty of modeling the oil change, the model presented an L-infinity norm error of 1 Newton, as shown in Figure 15A. This difference between the DNN model and real data could be explained by the noise present in the training measures, mainly the noise generated by the variation in the fluid volume during the multiple tests.

**Figure 16:**
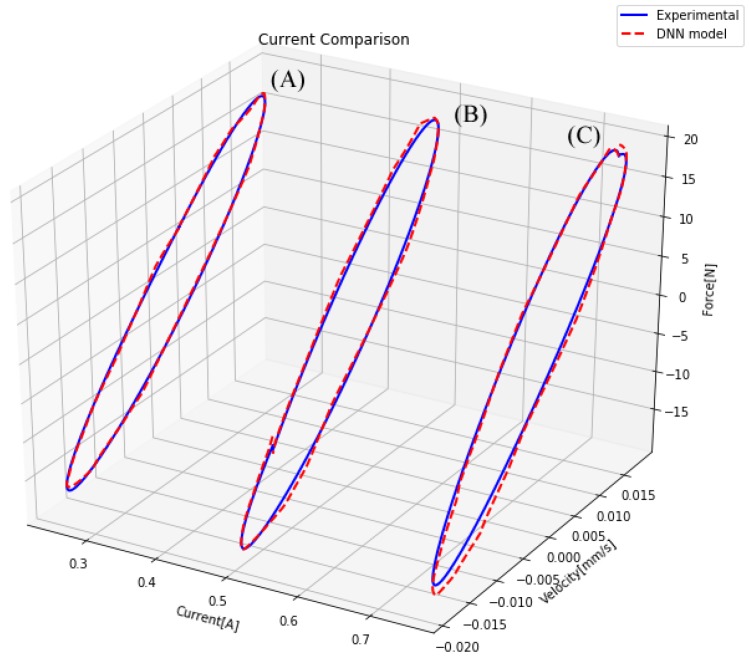
Comparison of a damper for three magnetic fields.

This model must be useful for performing controller design. For this reason, it must be capable of representing the damper behavior for different magnetic fields. In Figure 16, a comparison of the actual measures of the damper and the model approximations for the same damper with different magnetic fields is presented. In this comparison, it can be appreciated that the model performed in a similar way with regard to the real damper.

A statistical regression validation was performed to obtain a numerical quantification of the model performance. The measure of goodness of fit applied was the coefficient of determination $R^{2}$ [58]. The better is the regression, the closer is the value of $R^{2}$ to 1. In our case, the model performed with a coefficient of determination of $R^{2}=0.9577$.

## 5. Conclusions

A novel hybrid model that combines first-principles dynamic models with a phenomenological model based on a deep neural network is presented. This model is capable of representing the damper behavior when physical changes in geometric parameters are made and when it is subject to different magnetic fields. For this reason, the model can be used in a complete design methodology ranging from the damper geometry to the controller design. A methodology to develop non-parametric models of complex systems combining rapid prototyping with deep neural networks has been proposed. Such methodology takes advantage of additive manufacturing by obtaining physical measurements of many prototypes, as well as deep neural networks, which are capable of learning despite the noisy measurements caused by manufacturing imperfections among other reasons. The design methodology has been complemented with a detail explanation of the neural network design process, which should prove useful in applying the proposed methodology to other cases.

The ability of the proposed model was validated by experimental measures on a set of different conditions. The testing results indicate that the model could forecast the hysteretic responses of the magnetorheological damper for different load conditions and various physical configurations. Although the proposed methodology was successfully applied to a family of monotube dampers, it can be applied to any set of dampers as long as an adequate number of prototypes are generated throughout all the spectrum of possible solutions.

Finally, as shown in Table 2, the proposed model is the only one that can be used to analyze the response of the system and for the control and design tasks. In addition, it is distinguished by having physical design parameters as inputs to the model and at the same time considering the phenomenon of hysteresis as nonlinear. These characteristics show that the proposed model is very useful for future research in the area or technological developments.

## Figures and Tables

**Table 1:** Polynomial constants for different magnetic fluids.

Magnetic Fluid	$c_{0}$	$c_{1}$	$c_{2}$	$c_{3}$	$c_{4}$
122EG Mag. Pro.	7.003 × 10${}^{-15}$	233.446	−4.2465 × 10${}^{-14}$	205.765	5.413 × 10${}^{-14}$
122EG Yield S.	3.886	0.039	1.267 × 10${}^{-5}$	−6.876 × 10${}^{-9}$	0
140CG Mag. Pro.	0	73.468	1.0104 × 10${}^{-14}$	119.684	−1.778 × 10${}^{-14}$
140CG Yield S.	2.44053	0.5469	−0.00142579	5.357 × 10${}^{-7}$	0

**Table 2:** Magnetorheological models comparison.

Model Name	Type	Voltage	Design Parameters	Hysteresis	Reponse	Control	Design
Bingham model [51]	Parametric	No	No	No	Yes	No	No
Extended Bingham model [52]	Parametric	Yes	No	Linear Pre-yield	Yes	Partially	No
Bouc–Wen model [53]	Parametric	Yes	No	Linear	Yes	Partially	No
Modified Bouc–Wen model [31]	Parametric	Yes	No	Nonlinear	Yes	Yes	No
Chebyshev polynomial fit [54]	Non-parametric	Yes	No	Nonlinear	Yes	Partially	No
Neural networks [36]	Non-parametric	Yes	No	Nonlinear	Yes	Yes	No
Parallel-plate models [55]	First Principles	Yes	Yes	No	Yes	No	Yes
Dynamic Fluid Model [56]	First Principles+Parametric	Yes	Partially	Nonlinear	Yes	Partially	Yes
Multiphysics Model [57]	First Principles+Simulation	Yes	Partially	Linear	Yes	Partially	Yes
**Proposed Deep neural network**	**Non-parametric**	Yes	**Yes**	**Nonlinear**	Yes	**Yes**	Yes
